# Persistence of viral RNA in lymph nodes in ART-suppressed SIV/SHIV-infected Rhesus Macaques

**DOI:** 10.1038/s41467-021-21724-0

**Published:** 2021-03-05

**Authors:** Anthony M. Cadena, John D. Ventura, Peter Abbink, Erica N. Borducchi, Hubert Tuyishime, Noe B. Mercado, Victoria Walker-Sperling, Mazuba Siamatu, Po-Ting Liu, Abishek Chandrashekar, Joseph P. Nkolola, Katherine McMahan, Nicole Kordana, Venous Hamza, Esther A. Bondzie, Emily Fray, Mithra Kumar, Stephanie Fischinger, Sally A. Shin, Mark G. Lewis, Robert F. Siliciano, Galit Alter, Dan H. Barouch

**Affiliations:** 1 Center for Virology and Vaccine Research, Beth Israel Deaconess Medical Center, Harvard Medical School, Boston, MA 02215 USA; 2 Department of Medicine, Johns Hopkins University School of Medicine, Baltimore, MD 21205 USA; 3 Ragon Institute of MGH, MIT, and Harvard, Cambridge, MA 02139 USA; 4 Bioqual, Rockville, MD 20852 USA

**Keywords:** Cellular immunity, Viral reservoirs

## Abstract

The establishment of a long-lived viral reservoir is the key obstacle for achieving an HIV-1 cure. However, the anatomic, virologic, and immunologic features of the viral reservoir in tissues during antiretroviral therapy (ART) remain poorly understood. Here we present a comprehensive necroscopic analysis of the SIV/SHIV viral reservoir in multiple lymphoid and non-lymphoid tissues from SIV/SHIV-infected rhesus macaques suppressed with ART for one year. Viral DNA is observed broadly in multiple tissues and is comparable in animals that had initiated ART at week 1 or week 52 of infection. In contrast, viral RNA is restricted primarily to lymph nodes. Ongoing viral RNA transcription is not the result of unsuppressed viral replication, as single-genome amplification and subsequent phylogenetic analysis do not show evidence of viral evolution. Gag-specific CD8+ T cell responses are predominantly observed in secondary lymphoid organs in animals chronically infected prior to ART and these responses are dominated by CD69+ populations. Overall, we observe that the viral reservoir in rhesus macaques is widely distributed across multiple tissue sites and that lymphoid tissues act as a site of persistent viral RNA transcription under conditions of long-term ART suppression.

## Abstract (web summary)

The existence of HIV reservoir and ongoing replication despite antiretroviral therapy (ART) represents a barrier for cure efforts. Here, using SIV/SHIV-infected rhesus macaque suppressed with ART for one year, the authors characterize multiple lymphoid and non-lymphoid tissues and show that while the viral reservoir exhibits a wide anatomic heterogeneity, persistent viral transcription is mainly restricted to secondary lymphoid organs.

## Introduction

The key barrier for an HIV-1 cure is the establishment of a long-lived reservoir of latently infected resting memory CD4 T cells^1–5^. While various strategies are being explored to target the reservoir^6–14^, the rapid seeding and long half-life of the viral reservoir hampers current HIV-1 cure efforts^15–17^. Targeting the viral reservoir requires a comprehensive understanding of both the cellular populations and tissue sites associated with reservoir persistence. Recent studies have provided evidence that the HIV-1 viral reservoir is both cellularly and anatomically heterogeneous and that reservoir persistence is driven by the proliferation of clonal populations within and between anatomical compartments^18,19^. In addition, replication-competent reservoirs were detected across multiple tissue-sites in patients with the peripheral blood and secondary lymphoid organs (SLOs) the primary sources of rebounding virus^18^. As illustrated by these reports, how the anatomical distribution of the viral reservoir impacts not only viral rebound and reservoir diversity but also persistent viral transcription and tissue-specific cellular immune responses in both SIV-infected macaques^20–25^ and HIV-1-infected individuals^26–34^ during long-term antiretroviral therapy (ART) remain critically important questions.

To address these questions, we evaluated the virologic and immunologic properties of the viral reservoir in multiple tissues from long-term ART suppressed (≥1 year) SIVmac251 and SHIV-SF162P3 infected rhesus macaques. ART was initiated during both acute (Early ART group) and chronic (Late ART group) infection for a comprehensive evaluation of the viral reservoir in both settings. Gastrointestinal, reproductive, and SLOs were collected following a terminal necropsy performed during ART suppression and were assessed for the presence of cellular viral DNA and viral RNA by performing cell-associated SIV/SHIV quantitative PCR (qPCR) and SIV quantitative RT-PCR (qRT-PCR) assays, respectively^15,16^. To characterize antigen-specific T cell responses, we measured cellular cytokine responses via multiparameter flow cytometry^12,16^. Humoral immune responses were detected using SIV/SHIV Env-specific ELISA and subsequent systems serology analysis^35–37^. We observed a wide distribution of viral DNA expression across multiple tissues. By contrast, viral RNA expression was more anatomically restricted to lymph nodes. Virus-specific CD8**+** T cell responses were predominately observed in secondary lymphoid tissues, primarily detected in CD69+ populations, and were higher in animals that were chronically infected prior to ART initiation. Tissue-specific humoral responses strongly correlated with the extent of viral replication prior to ART initiation, with broader antibody functionality found in the early ART versus the late ART cohort. These data show that although the viral reservoir exhibits substantial anatomic heterogeneity, persistent viral transcription is mainly restricted to SLOs.

## Results

### Cell-associated SIV/SHIV RNA is persistently expressed in lymphoid tissues during long-term suppressive ART

**Fig. 1:**
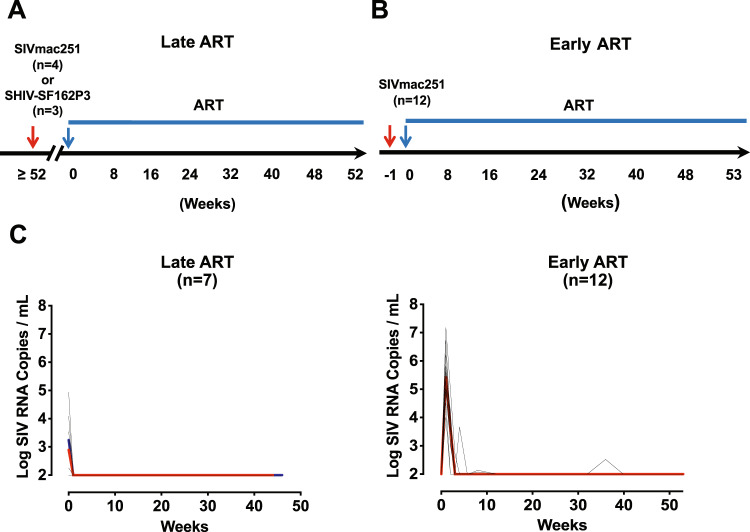
Experimental design of Late and Early ART-suppressed SIV or SHIV-infected cohorts. **A** Late ART study design: 7 rhesus monkeys were infected with either SIVmac251 or SHIV-SF162P3 (*n* = 4 and 3 animals/group, respectively) for at least a year (≥52 weeks). ART was initiated at week 0 and daily ART administration was maintained until the day of necropsy. **B** Early ART study design: 12 rhesus monkeys were infected with SIVmac251 for 1 week. Animals were maintained on daily ART suppression until the day of necropsy. **C** Plasma viral loads are shown for the Late ART (median line for SIVmac251 infected animals in red and median line for SHIV- SF162P3 infected animals in blue) and Early ART studies. Log SIV-RNA copies/ml are shown (LOD = 50 RNA copies/ml). Black lines indicate individual monkey values. ART, antiretroviral therapy.

We performed detailed necropsies on 19 rhesus macaques (*Macaca mulatta)* infected with SIVmac251 or SHIV-SF162P3 and suppressed with ART for approximately 1 year (42–56 weeks). ART was initiated either during chronic infection (Late ART, n = 7) or during acute infection (Early ART, n = 12) (Fig. 1A, B). In the Late ART study, 4 monkeys were infected with 5 × 10^4^ TCID_50_ SIVmac251 over 12 months prior to initiation of daily subcutaneous administration of a triple-drug ART cocktail (tenofovir disproxil fumarate, emtricitabine, and dolutegravir) and 3 monkeys were infected with 5 × 10^4^ TCID_50_ SHIV-SF162P3 over 12 months prior to initiation of daily ART^9,12^. In the Early ART study, 12 monkeys were infected with 5 × 10^4^ TCID_50_ SIVmac251 7 days prior to initiation of ART^9^. All animals were fully suppressed on ART at the time of necropsy (Fig. 1C). Cell-associated viral DNA and viral RNA were quantitated from 24 different tissue sites using cell-associated *gag* qPCR and qRT-PCR, respectively^16,38^. These tissues included the female reproductive tract, the gastrointestinal (GI) tract, draining and distal lymph nodes, secondary lymphoid organs, lung, liver and the central nervous system. Negative and positive control animals included 3 uninfected animals and 1 viremic SIVmac251-infected animal, respectively (Supplementary Fig. 1).

**Fig. 2:**
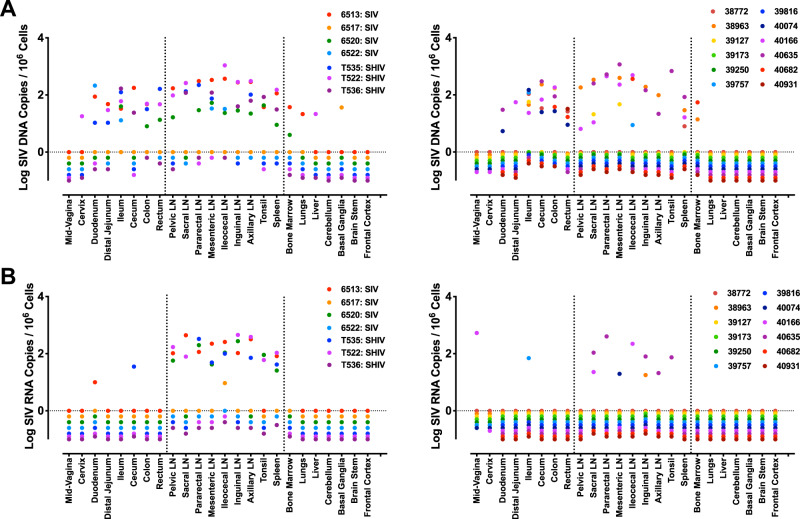
Viral DNA and viral RNA in tissue necropsies from Late and Early ART-suppressed rhesus monkeys. **A** Copies of total viral DNA and **B** viral RNA from 24 tissue necropsies from the Late ART (left) and Early ART (right) studies. Log SIV DNA copies/10^6^ cells and log SIV RNA copies/10^6^ cells are shown. Colors reflect individual animals. Assay sensitivity was >8–10 SIV DNA copies/10^6^ cells and 10 SIV RNA copies/10^6^ cells. SIV, simian immunodeficiency virus.

Following approximately 1 year of ART suppression, viral DNA (median 1.74 log viral DNA copies per 10^6^ cells) was broadly detected in various tissues, including multiple SLOs and GI tissues in both the Late and Early ART studies (Fig. 2A). These findings are consistent with previous descriptions of chronic SIV/SHIV infection^24,39,40^ and acute SIV infection, both with and without ART^16,24,41,42^. Low levels of viral DNA were also detected in the cervix, lungs, liver and brain in a subset of animals. 100% (7 of 7) of the Late ART monkeys had at least one SIV DNA positive tissue, whereas 75% (9 of 12) of the Early ART monkeys had detectable levels of viral DNA (Supplementary Fig. 2). Levels of viral DNA were comparable in both the Late and Early ART studies (median 1.69 and 1.84 log viral DNA copies per 10^6^ cells, respectively), suggesting that initiation of ART at 1 week did not result in lower viral DNA compared with initiation of ART at 1 year (Supplementary Figs. 3 and 4A). Levels of viral DNA also appeared comparable in SIV and SHIV-infected monkeys in the Late ART study. Moreover, levels of viral DNA were comparable in lymphatic and GI tissues (Supplementray Fig. 5A and Supplementary Table 1). Overall, these data demonstrate that viral DNA was found broadly in multiple tissues, particularly in secondary lymphoid and GI tissues, over the course of 1 year of ART suppression.

**Fig. 3:**
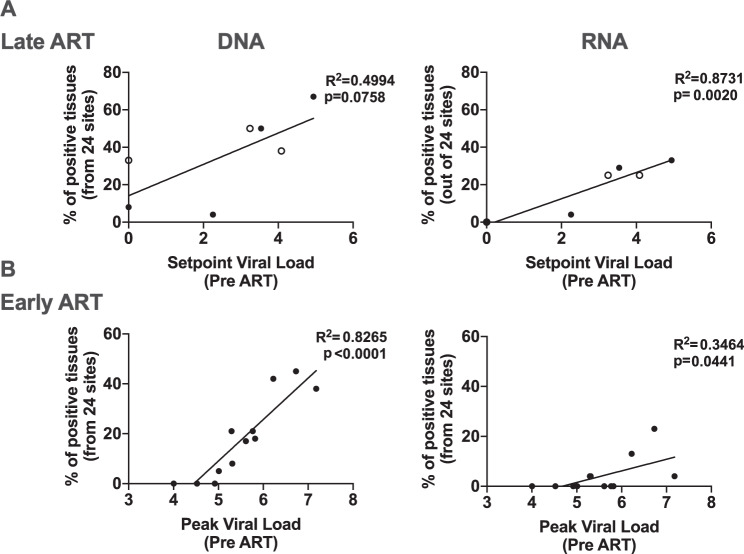
Associations between pre-ART viral loads and tissue-associated peak viral loads. **A** Linear relationships between pre-ART plasma viral loads and tissue positivity for the (**A**) Late ART and (**B**) Early ART monkey cohorts. The setpoint viral loads at week 0 and the peak viral loads at week 1 were used for the Late ART and Early ART linear regressions, respectively. Each circle is a monkey and open circles denote SHIV-SF162P3 infected animals in the Late ART study. R squared and p values were derived using a simple linear regression model.

Viral RNA (median 1.37 log viral RNA per 10^6^ cells) was observed in lymphoid tissues in both the Late (71%, 5 of 7) and Early (33%, 4 of 12) ART animals (Fig. 2B). These findings are consistent with recent reports of persistent viral RNA despite ART treatment in monkeys and humans^24,40,43–49^. Remarkably, in contrast to the broad tissue distribution observed for viral DNA, viral RNA was almost exclusively detected in lymph nodes (Fig. 2B**)**. Similar levels of total viral RNA copies were observed between the Late and Early ART studies (median 2.02 and 1.87 log viral RNA per 10^6^ cells, respectively) as well as between the SIV- and SHIV-infected animals (Supplementary Figs. 3 and  4). Only minimal viral RNA was detected in GI tissues and other non-lymphoid tissues, despite robust levels of viral DNA in these tissues (Fig. 2, Supplementary Fig. 5 and Supplementary Table 1). Moreover, the relationship between the number of positive tissues for both viral DNA and viral RNA was highly linear with pre-ART viral loads in both the Late and Early ART studies (Fig. 3). Taken together, these data show that persistent transcription of viral RNA could be detected in animals that had initiated ART during both acute and chronic infection despite 1 year of ART suppression, and viral transcription was restricted almost exclusively to lymphoid tissues.

**Fig. 4:**
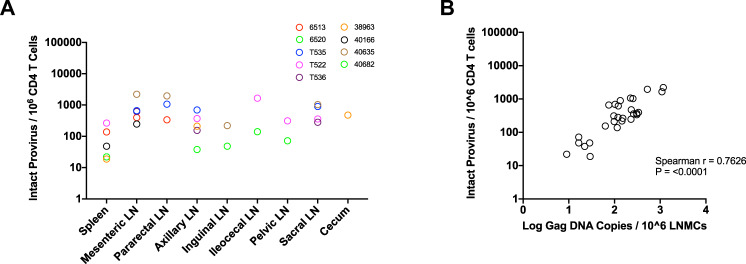
Distribution of Intact Proviral sequences per 10^6^ CD4 T cells in lymphoid tissues from animals in the Late ART and Early ART rhesus monkeys. **A** Tissue distribution of intact proviruses per 10^6^ CD4 T cells from rhesus macaques in the Late and Early ART cohorts measured by the SIV Intact Proviral DNA Assay (IPDA). Each colored dot represents one animal. **B** Correlation between IPDA viruses measured in each lymphoid tissue selected in (**A**) and viral DNA *gag* copies per 10^6^ lymph node mononuclear cells (LNMCs) (Fig. 2).

Quantitative PCR assays that detect SIV proviruses by amplifying conserved sequences in *gag* can be highly sensitive and reproducible, but are subject to overrepresentation of defective proviral sequences and underrepresentation of intact proviral sequences. As intact proviruses would comprise the source of infectious virus upon ART interruption, (i.e., the replication-competent reservoir) we sought to quantify and determine the distribution of intact proviruses across multiple tissue sites from the Late and Early ART cohorts. Intact proviral sequences were quantified using the recently published Intact Proviral DNA Assay (IPDA), a multiplexed digital droplet PCR (ddPCR) assay designed to detect intact and non-hypermutated SIV and SHIV proviral sequences^50^. Intact proviral sequences per 10^6^ CD4 T cells ranged greatly between different animals as well as different tissue sites (Fig. 4A, Supplementary Fig. 6), and correlated strongly with viral DNA copies measured via qPCR (Fig. 4B, Spearman r = 0.7626, p = <0.001). Notably, the number of intact proviruses within each animal varied broadly between 22.0 to 71.9 copies per million (animal 6520) and 219.7 to 2208.2 copies per million (animal 40635) when accounting for all tissues analyzed (Supplementary Fig. 6).

### CD69^+^CD8^+^ T cell responses persist in secondary lymphoid organs and depend on the timing of ART initiation

**Fig. 5:**
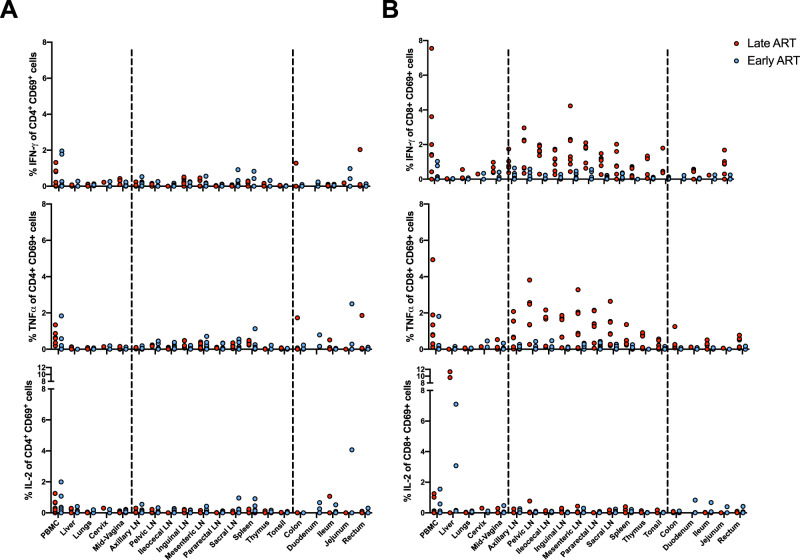
T lymphocyte cytokine responses in ART-suppressed tissues. **A**, **B** SIV-Gag specific IFN-γ, IL-2, and TNFα responses in CD69 + CD4 + (**A**) and CD8 + (**B**) T cells at necropsy following 1 year of ART for the Late ART monkeys (red dots) and Early ART monkeys (blue dots). Colored dots refer to individual animals. All samples with approximately 2,000 or more CD69 + cells were kept for further analysis. Flow cytometry gating strategy used is illustrated in Supplementary Fig. 7. IFN-γ, Interferon gamma, IL-2, Interleukin 2, TNFα, Tumor necrosis factor alpha.

To better understand why persistent cell-associated SIV/SHIV transcription was restricted to lymphatic tissues, we evaluated SIV/SHIV gag-specific CD4 + and CD8 + T cell immune responses by performing multiparameter intracellular cytokine staining (ICS)^16,51,52^. We assessed CD4+ and CD8+ T cell responses expressing IFN-γ, IL-2, and TNFα (Fig. 5, Supplementary Figs. 7–10). In both the Late ART and Early ART groups, the highest levels of virus-specific T cell responses were observed in lymph nodes, the rectum, and the peripheral blood, with lower levels observed in the female reproductive tract, the upper GI tract, other non-lymphoid tissues (Fig. 5). Virus-specific responses were elicited by CD69+ T cells, suggesting that in tissues, these cells were predominantly expressing a T_RM_ phenotype (Supplementary Fig. 10). Virus-specific CD69^+^CD8^+^ T cell responses were higher in lymph nodes than other tissue sites (Fig. 5B, Supplementary Fig. 8B). The frequency of virus-specific T_H_1 CD4^+^ T cell responses were lower than CD8^+^ T cell responses, but were still detectable in the peripheral blood, lymph nodes, spleen, and female reproductive tract (Fig. 5A, Supplementary Figs. 8A and 9A). CD8^+^ T cell responses were higher in animals that were chronically infected prior to the initiation of ART, most likely reflecting the short duration of virus replication in animals in the Early ART cohort (i.e., 7 days) (Fig. 5, Supplementary Figs. 7 and 8). Notably, virus-specific CD69^+^CD8^+^ T cell responses were primarily localized to lymph nodes, which were the same anatomical sites exhibiting detectable low-level viral transcription. Taken together, these data show that chronic SIV/SHIV infection generates robust CD69^+^CD8^+^ T cell responses in lymph nodes, and that these responses are maintained throughout long-term suppressive ART, likely in response to persistent viral RNA in lymph nodes.

### Functional humoral responses in serum and tissues depend on the timing of ART initiation

**Fig. 6:**
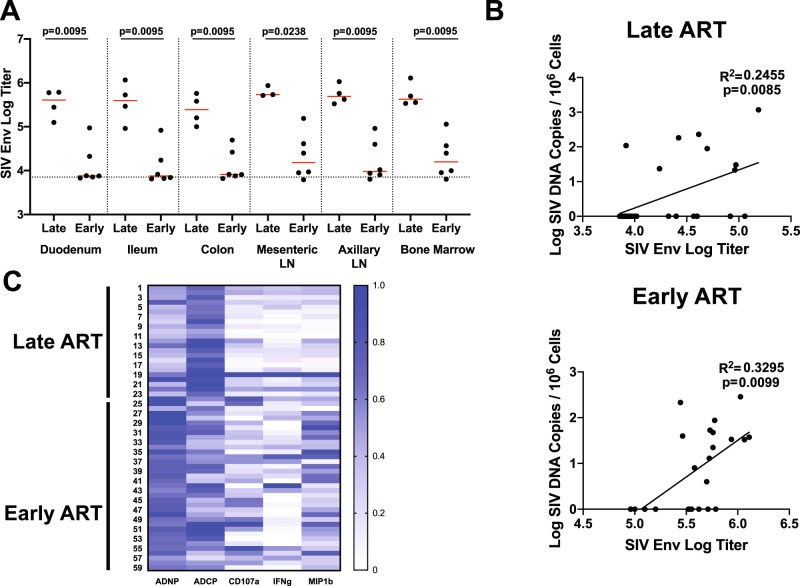
Humoral responses in ART-suppressed tissues. **A** SIVmac23H Env titers from multiple gut and LTs from a subset of SIV-infected monkeys from both the Late ART (*n* = 3 for mesenteric LN and *n* = 4 for all other tissues) and Early ART studies (*n* = 6). The median is shown as a red line and p values reflect two-tailed Mann Whitney U tests. **B** Linear regression of SIVmac32H Env titers (x axis) and log SIV DNA copies/10^6^ cells (y axis) in the profiled tissues in the Late ART (upper) and Early ART (lower) studies. **C** The Fc functional profiles of these SIV Env antibodies are shown for the Late ART and Early tissues. Each row in the heat map is a different tissue Fc profile for antibody dependent neutrophil phagocytosis (ADNP), antibody dependent cellular phagocytosis (ADCP), and NK functionality (CD107a/IFN-γ/MIP1β expression). LN, lymph node, Env, Envelope.

We next evaluated Env-specific humoral immune responses in tissues using ELISA and systems serology^35–37^. As expected, SIV-infected monkeys in the Late ART study had elevated levels of serum anti-SIV Env titers (as assessed by binding to SIVmac23H antigen) compared to the SIV-infected monkeys in the Early ART study (Supplementary Fig. 11). We assessed anti-SIV Env titers in a subset of tissues from monkeys from both studies using tissue supernatants from necropsy. We found comparable anti-SIV Env titers in cell supernatants from GI tissues and lymphoid tissues, and Env titers were markedly higher in the Late ART study compared with the Early ART study (Fig. 6A). Moreover, in both the Late ART and Early ART studies, anti-Env titers were associated with levels of SIV DNA, suggesting that increased viral replication led to augmented antibody responses (p = 0.0085 and p = 0.0099, respectively) (Fig. 6B). Fc functionality of these Env-specific antibody responses differed between the Late and Early ART studies (Fig. 6C). In monkeys that initiated ART after 1 year of infection, the primary functionality of these tissue antibodies as antibody-dependent cellular phagocytosis (ADCP) (Fig. 6C). In contrast, in monkeys that initiated ART after 1 week, we observed a broader functional profile characterized by neutrophil and macrophage phagocytosis as well as Natural-Killer Cell (NK) activity (Fig. 6C). These data suggest that the magnitude and functionality of the antibody response largely reflected the extent of viral replication and the time of ART initiation.

### Viral RNA in lymph nodes does not show evidence of viral evolution

**Fig. 7:**
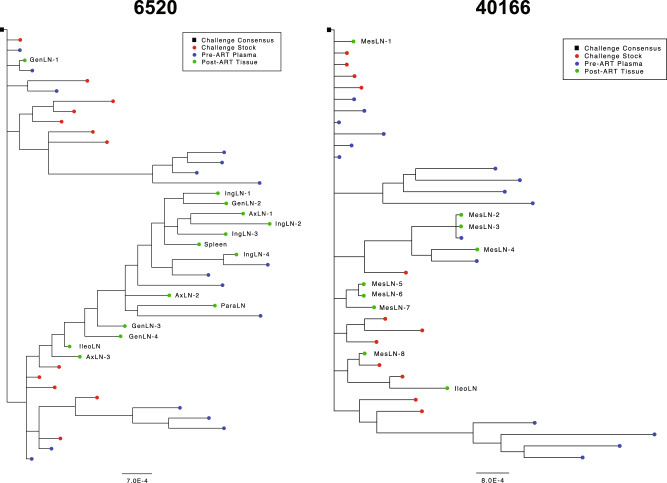
Phylogenetic analysis of the *gag-pol* sequences of macaque 6520 and 40166. *Gag-pol* sequences from SIVmac251 infected animals 6520 (Late ART cohort) and 40166 (Early ART cohort) isolated by single genome amplification (SGA). Viral sequences were sampled at challenge (in red), were derived from pre-ART plasma (in blue), and from lymph nodes at necropsy during ART suppression (in green). The root of each tree is the consensus challenge stock (black square). Branch length distance defined as number of mutations per base. MesLN, mesenteric LN, GenLN, genital LN, IngLN, inguinal LN, AxLN, axillary LN, ParaLN, pararectal LN.

To assess whether persistent viral RNA in lymphoid tissues was due to low levels of viral replication, we sequenced a region intersecting *gag* and *pol* by single genome amplification (SGA) and performed a phylogenetic analysis^50,53^. Using SGA on four representative monkeys from both study cohorts, we found no evidence of viral evolution in the *gag-pol* sequences following 1 year of ART compared with pre-ART sequences (Fig. 7, Supplementary Fig. 12). For all animals sequenced, pre-ART viral sequences derived from plasma occupied clades that also included sequences sampled at necropsy. These data suggest that the persistent viral RNA in lymphoid tissues likely does not reflect ongoing virus replication and subsequent genetic diversification, but rather is derived from cell-associated transcription of proviral RNA from within the reservoir. Notably, we detected identical sequences from mesenteric lymph nodes in animal 40166 as well as identical sequences between the mesenteric lymph node and plasma compartments, perhaps resulting from a clonally expanded memory population (Fig. 7). Whether clonal expansion occurs in macaques under long-term suppression at the same level as in humans ART suppressed for many years is still an open question and additional evidence is required to show if clonal expansion is indeed occurring within these tissue sites after one year of ART suppression in macaques.

### Persistent viral RNA expression does not reflect insufficient duration of suppressive ART

**Fig. 8:**
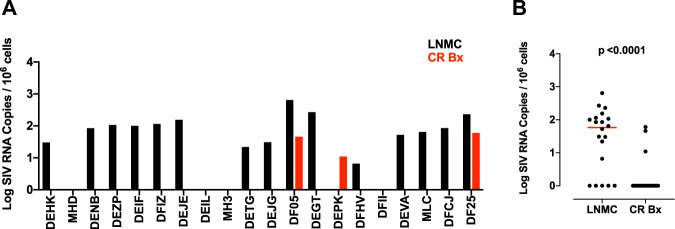
Viral RNA in lymph node and colorectal biopsies of long-term ART suppressed monkeys. **A** Log SIV RNA copies/10^6^ cells are shown from lymph node mononuclear cells (LNMCs) derived from lymph node tissue (black bars) and colorectal biopsies (red bars) from 20 rhesus monkeys chronically infected with SHIV-SF162P3 and ART-suppressed for 3 years. Each bar represents the SIV viral load of the monkey listed in the indicated tissue site as the log of SIV RNA copies/10^6^ cells. **B** Viral RNA persistence in lymph nodes from monkeys under 3 years of ART suppression. Each dot is a monkey (*n* = 20). The median is displayed as a red line. p value is derived from a two-tailed Mann-Whitney U test. LNMC, lymph node mononuclear cells, CR Bx, colorectal biopsy.

Finally, we addressed the possibility that the persistent viral RNA in lymph nodes following 1 year of ART suppression may simply reflect an insufficient period of time on suppressive ART. Our laboratory and others have shown that a substantial period of time can be required for prolonged virologic suppression^9,54,55^. To evaluate whether a longer period of ART suppression would fully suppress viral RNA in lymph nodes, we treated a separate cohort of 20 chronically SHIV-SF162P3-infected rhesus macaques with daily suppressive ART for 3 years. Afterward, viral RNA in inguinal and axillary lymph nodes and colon biopsies were evauated. Viral RNA was observed in 75% (15 of 20) animals in lymph nodes (median 1.77 log viral RNA per 10^6^ cells) (Fig. 8), which is comparable to the frequency and levels of viral RNA observed in animals after 1 year of suppressive ART (Fig. 2). Moreover, viral RNA was detected only sporadically in gastrointestinal biopsies in animals after 3 years of suppressive ART (Fig. 8). These data demonstrate that similarly to animals suppressed for 1 year on ART, viral RNA expression in SHIV-SF162P3-infected rhesus macaques is restricted to lymphoid tissues despite 3 years of suppressive ART, and that persistent viral RNA transcription is not predominantly a result of insufficient time on suppressive ART.

## Discussion

In this study, we provide a comprehensive analysis of the anatomic features of the viral reservoir in SIV/SHIV-infected rhesus macaques after long-term suppressive ART. We observed extensive anatomic distribution of viral DNA, with comparable levels of viral DNA in animals that initiated ART at week 1 and week 52 of infection. In addition, we show persistent viral RNA in lymph nodes and that viral transcription was associated with durable virus-specific CD69^+^CD8^+^ T cell populations in lymph nodes. These data suggest that the lymphoid reservoir is not transcriptionally silent despite 1–3 years of ART suppression, and that ongoing viral transcription may help maintain CD69^+^CD8^+^ T cell responses in lymph nodes. These findings have important implications for HIV-1 cure strategies that aim to target and eliminate this viral reservoir.

The broad tissue distribution of viral DNA despite suppressive ART is consistent with the known stability of the viral reservoir in both monkeys and humans^1,2,15,40,56,57^. Viral DNA was less frequent but similar in magnitude in the Early ART study compared with the Late ART study, consistent with recent reports showing the reservoir is seeded very early following infection^15,55,58^. If these findings translate to HIV-1-infected humans, they question the widely held assumption that it will necessarily be easier to achieve an HIV-1 cure in acutely-treated compared with chronically-treated HIV-1-infected individuals, although viral diversity will likely be lower in acutely treated individuals.

Interestingly, a prior report suggested that the GI tract may be the primary repository of viral RNA^24^. Differences between that report and our findings could perhaps be explained by the longer duration of ART in the present study. Additional studies have reported that HIV-1-infected individuals on ART for many years still had detectable viral RNA in lymph nodes, consistent with our findings^44^. Sequencing data from that report suggested that viral RNA resulted from clonal proliferation rather than viral replication. Our data are consistent with these findings and we did not detect any increase in viral diversity from our phylogenetic analysis.

Viral RNA persistence in lymphoid tissues despite 1 year (Fig. 2) or 3 years (Fig. 8) of suppressive ART has important implications for our understanding of reservoir biology and for the development of HIV-1 cure strategies. These data demonstrate that there are key anatomic differences in the viral reservoir and show that a small fraction of reservoir cells in lymph nodes are transcriptionally active, supporting recent reports of persistent viral RNA in both monkeys^24,43^ and humans^44^. We speculate that transcriptionally active reservoir cells may occasionally produce sufficient virus to result in viral “blips” despite ART, and thus do not indicate antiretroviral drug resistance or impending virologic failure.

The mechanism underlying the persistent viral RNA in lymph nodes remains to be determined. We observed persistent CD69^+^CD8^+^ T cell responses in lymph nodes, particularly in the Late ART group, suggesting that this population may be responding to SIV/SHIV antigen expression in lymph nodes. Previous reports have shown that up to 40–80% of all CD8^+^ T cells in lymph nodes from HIV-1 infected patients had a canonical T_RM_ phenotype and that the majority of CD8^+^ T_RM_ in those lymph nodes were HIV-1-specific^59^. It is possible that SIV-specific CD8^+^ T_RM_ cells may exhibit a shift to a more “helper-like” polyfunctional effector phenotype with greatly attenuated cytolytic capacity in natural infection, as shown in recent reports for HIV-1 progresses in elite controllers^59–61^.

In summary, our data support a model in which the tissue viral reservoir is rapidly and broadly seeded early during acute infection and that viral RNA persists in lymph nodes and other secondary lymphoid tissues despite 1–3 years of suppressive ART. Therefore, viral latency does not appear to be universally transcriptionally silent, and the reservoir may include a spectrum of latency depths. Transcriptional activity of the lymphoid viral reservoir could potentially be exploited by immunologic strategies that aim to target the reservoir, assisting in the development of next generation HIV-1 cure strategies.

## Methods

### Ethics statement

All studies involving monkeys in this manuscript were conducted in accordance with and with the approval of the Bioqual Institutional Animal Care and Use Committee (IACUC). Rhesus macaques were housed at the Bioqual Animal Facility (Bioqual, Rockville, MD) and daily care was carried out by certified and professional animal welfare and veterinary staff according to the Facility’s guidelines for animal handling and care.

### Animals

A total of 44 outbred, Indian- origin adult or juvenile female or male rhesus monkeys (*Macaca mulatta*) were selected and housed at the Bioqual Animal Facility (Bioqual, Rockville, MD). Monkeys were randomly assigned between two experimental study groups, Early ART treatment and Late ART treatment. A negative control and a positive control cohort of monkeys were designated as well. Monkeys were randomly allocated to groups based on sex. Monkeys expressing the major histocompatibility complex (MHC) Class I molecules Mamu-A*, Mamu-B*08, and Mamu-B*17 were not used in this study. *Trim5*a polymorphisms, which increase the ability of the virus to replicate, were distributed evenly between study groups.

### Late and early ART initiation

Eight female monkeys were included in the Late ART treatment study. Four monkeys were infected with 5 × 104 tissue culture infectious dose 50 (TCID_50_) of SIVmac251 and remained viremic for 2 years prior to initiation of preformulated ART consisting of tenofovir disoproxil fumarate, emtricitabine (reverse transcriptase inhibitors), and dolutegravir (integrase inhibitor) (TDF/FTC/DTG; Gilead). An additional 4 monkeys were infected with 5 × 10^4^ tissue culture infectious dose 50 (TCID50) of SHIV-SF162P3 and remained viremic for 1 year prior to initiation of ART (TDF/FTC/DTG; Gilead)^62^. One monkey (T521) infected with SHIV-SF162P3 died due to AIDS complications and was excluded from analysis. For the Early ART animal cohort, twelve monkeys acutely infected with 5 × 10^4^ tissue culture infectious dose 50 (TCID_50_) SIVmac251 for 7 days prior to initiation of preformulated ART consisting of tenofovir disoproxil fumarate, emtricitabine, and dolutegravir (TDF/FTC/DTG; Gilead)^62^. For both Late and Early ART cohorts, ART treatment was administered until the day of necropsy for each animal, which varied between 38 and 52 weeks depending on the animal.

### Control groups

Necropsies and virologic analyses were performed on several practice animals, 3 uninfected and untreated monkeys (*n* = 3) and 1 SIVmac251-infected but untreated monkey (*n* = 1).

### Necropsy

For each monkey in this study, 24 tissues were collected. These tissues included: Mid-Vagina, Cervix, Duodenum, Distal Jejunum, Ileum, Cecum, Colon, Rectum, Pelvic LN, Sacral LN, Pararectal LN, Mesenteric LN, Ileocecal LN, Inguinal LN, Axillary LN, Tonsil, Spleen, Bone Marrow, Lungs, Liver, Cerebellum, Basal Ganglia, Brain Stem, and Frontal Cortex. For each type of tissue, multiple samples were excised at necropsy by a board-certified pathologist. Tissues samples for vDNA and vRNA extraction were immediately flash frozen following excision to preserve nucleic acid integrity. The remaining samples were sent on ice the day of the necropsy for tissue processing and subsequent immunological analysis. Two necropsies were performed every week for a period of 12 weeks which translated into nearly a year of ART suppression (38–52 weeks).

### Long term ART suppression

An additional 20 rhesus macaques chronically infected with SHIV-SF162P3 (TCID50 of 5 × 10^4^) were treated with daily suppressive ART (TDF/FTC/DTG; Gilead)^62^ for 3 years. Viral RNA in lymph nodes mononuclear cells (LNMC) and rectal biopsies were evaluated after 3 years.

### Nucleic acid extraction & reverse transcription

Genomic DNA or total RNA were extracted from flash frozen tissues with a QIAcube HT (Qiagen, Germany) using the QIAamp 96 QIAcube HT kit or RNeasy 96 QIAcube HT kit. In brief, two small samples from each frozen tissue (approx. 30 mg) were cut and placed in separate, 2 mL Eppendorf tubes. Each tube held a 5 mm stainless-steel bead and either 700uL of QIAzol lysis buffer or 450uL of ATL buffer for RNA and DNA extraction, respectively. The tubes for total RNA extraction were then shaken twice at 25 Hz for 5 minutes using the Qiagen Tissue Lyser II (Qiagen, Germany). After lysis, 200uL of chloroform was added and manually shaken. Samples were incubated for 3 minutes at RT and then centrifuged for 15 minutes at 4 °C. After centrifugation, the aqueous phase was isolated and transferred to a deep-well, 96-well plate and total RNA was isolated using the QIAcube HT system. For DNA extraction, the tubes with tissue and ATL buffer were shaken twice for 1 minute at 25 Hz in the Qiagen Tissue Lyser II. 50uL of proteinase K was added to each sample and then samples were incubated overnight at 56 °C with shaking. Genomic DNA was extracted using the Qiacube HT system.

### Quantitative RT-PCR viral RNA and quantitative PCR viral DNA assays

A Quantitative RT-PCR assay was used to measure plasma viral loads and tissue-associated SIV RNA and a quantitative PCR (qPCR) viral DNA assay was used to determine cellular viral copies^63^. SIV *gag* RNA standards were generated using the AmpliCapMax™ T 7 High Yield Message Maker Kit (Cell Script) and purified with RNA clean and concentrator kit (Zymo Research, CA, USA). Log dilutions of the RNA or SIV *gag* standards were included with each RT-PCR assay and an RPP30 control was included in viral DNA qPCR assay. For RT-PCR, reverse transcription of both standards and samples was done using Superscript III VILO, following the manufactorer’s instructions (Invitrogen). RT-PCR assays were run in duplicate and qPCR assays were run in triplicate in 96 well MicroAmp Fast Optical plates (ThermoFisher) on the Quantstudio 6 Flex system (Applied Biosystems) using the following thermocycle settings: 95 °C for 20 s for initial denaturation, and 95 °C for 1 s followed by 60 °C for 20 s repeated for 45 cycles. All primers used are listed in Supplementary Table 2. For the qPCR assay, *RPP30* specific primers and probes were used from the *RPP30* copy number assay Hs00922551_cn (ThermoFisher, #4400291). Plasma viral loads were calculated as viral RNA copies per mL with an assay sensitivity of 50 copies per ml. Tissue qPCR for DNA copies/million cells were calculated using the *RPP30* gene to back calculate for cell input. The tissue qPCR DNA assay sensitivity was 8 copies per million cells. Tissue viral loads for RNA were first calculated as RNA copies per μg of total RNA input and then converted to RNA copies per million cells using the conversion factor of 10 pg = 1 mammalian cell^64,65^. Viral RNA assay sensitivity was 1 copy per μg of total RNA input (10 copies per million cells).

### Digital droplet PCR intact proviral DNA assay (IPDA)

The intact proviral DNA assay (IPDA) was used to determine the number of intact SIV proviruses^50^. In brief, total CD4^+^ T cells were isolated from frozen tissue single-cell suspensions using a Non-Human Primate negative magnetic bead separation kit (STEMCELL Inc.) according to the manufacturer’s instructions. Total genomic DNA (gDNA) was extracted from isolated CD4^+^ T cells using the QIAamp DNA mini (Qiagen) following the manufacturer’s instructions. The IPDA consists of three ddPCR reactions: (1) IPDA *pol* and *env* for determining intact proviruses, (2) 2’LTR for determining 2-LTR circle counts, and (3) *RPP30* for determining cell equivalents and the DNA shearing index. For the IPDA and 2-LTR reactions, 5.5 μl of gDNA (~800 ng) was added to a master mix containing 10 μl of 2X ddPCR Supermix (Bio-Rad), 600 nM of each primer and 200 nM of each probe per reaction volume totaling 22 μl. Input cell equivalents were calculated by amplifying the *RPP30* housekeeping gene and 3 ng of gDNA was used as input for *RPP30* controls. A shearing Index was calculated to account for the level of DNA fragmentation between two *RPP30* probe sites the same distance apart as the *pol* and *env* sites for the IPDA reaction. Shearing indices were calculated and the number of intact proviruses were determined by subtracting the number of 2LTR circles and normalizing the result to copies per million cells^50^. Primer and probe sequences are described in Supplementary Table 2 and were adapted from Bender et al. and Policicchio et al.^50,66^ . IPDA, 2-LTR, and RPP30 Reactions underwent the following cycling conditions: 10 minutes at 95 °C, 30 s at 94 °C and 2 min at 56 °C repeated for 50 cycles, 10 minutes at 98 °C, 10 minutes at 4 °C, with a final hold at 4 °C. All results were analyzed by QuantaSoft Analysis Pro software Vr. 10.596 (Bio-Rad) and samples that had at least 2 technical replicates with droplet counts >10,000 for each ddPCR reaction and a shearing index below 0.25 were kept for further analysis.

### Multiparameter Intracellular Cytokine Staining

Multiparameter intracellular cytokine staining (ICS) assays were performed as previously described^51,52,62^. A 10-color ICS assay was designed containing the following volume of monoclonal antibodies (Abs) per 100uL staining reaction: 0.25uL CD3 (SP34.2, Alexa700, BD #557917), 1.25uL CD4 (L200, V500, BD #561488), 1.25uL CD8 (SK1, APC-Cy7, BD #557834), 1uL CD69 (TP1.55.3, ECD, Beckman Coulter #6607110), 10uL CD28 (L293, PerCP-Cy5.5, BD #337181), 1uL CD95 (DX2, PE, BD #555674), 2.5uL IFNγ (B27, PE-Cy7, BD #557643), 0.25uL IL-2 (MQ1-17H12, APC, #554567) and 0.25uLTNFα (Mab11, FITC, #554512). Cells were gated on singlets, lymphocytes, live or dead lymphocytes, CD3, CD4 and CD8, CD69, and the pro-inflammatory cytokines TNFa, IFNg, and IL-2. Frequencies of activated CD4 + T cells in tissues are determined using a threshold of 2,000 CD69^+^ T cells. Tissues with less than 2,000 CD69^+^ T lymphocytes frequency were excluded from analysis. A negative control of R10 media and a positive control of phorbol myristate acetate (PMA) mixed with ionomycin, which stimulates the production of cytokines, were used to compare and gate for experimental immune response cytokine signatures of all monkeys.

### ELISA

SIV-specific humoral immune responses were assessed by SIVmac32H Env ELISAs, as previously described^62^. In brief, 96 well Maxisorp ELISA plates (Thermo Fisher Scientific) coated overnight with 100 ng/well of SIV mac32H gp140 Env in PBS were blocked for 2 hours with Casein in PBS Blocker (Thermofisher). NHP sera were then added in serial dilutions and incubated for 1 hour at room temperature. The plates were washed three times with PBS containing 0.05% Tween 20 and incubated for 1 hour with a 1/1000 dilution of HRP-conjugated goat anti-human secondary antibody (Jackson ImmunoResearch Laboratories). The plates were washed three times and developed with SureBlue tetramethylbenzidine (TMB) microwell peroxidase (KPL Research Products), stopped by addition of stop solution (KPL Research products) and analyzed at 450 nm on a Versamax microplate reader (Molecular Devices) using Softmax Pro 6.1 software. ELISA endpoint titers were defined as the highest reciprocal serum dilution that yielded absorbance > 2-fold background and absolute optical density (O.D.) of >0.2.

### Luminex isotyping and subclassing

For the measurement of relative subclass and isotype titers, a customized Luminex subclassing assay was used^67^. Fluorescent, carboxylated beads (Luminex) were coupled with SIVmac gp140 and Ebola gp (Immune tech) as a control antigen. Coupling was performed as a two-step reaction by covalent N-hydroxysuccinimide (NHS)–ester linkages via EDC (Thermo Scientific) and Sulfo-NHS (Thermo Scientific)^67^. 5 ul of 1:10, 1:100, and 1:1000 diluted sample in PBS was added to a 384 well plate (Greiner Bio-One). Per well, 1.2 × 10^3^ beads per Luminex region were added in Luminex assay buffer containing 0.1% BSA and 0.05% Tween-20. Microspheres and samples were incubated for 16–18 hours at 4 °C while shaking at 900 rpm. After incubation, microspheres were washed three times with 60 μl of Luminex assay buffer with an automated plate washer (Tecan). PE-coupled IgG1, IgG2-, IgG3-, IgG4, IgA1-, IgA2-, IgM- or bulk IgG-specific detection reagents (Southern Biotech) were added and incubated with immune complexes for 1 h at room temperature while shaking at 900 rpm. The coated beads were then washed and read on the iQue (Intellicyt). Bead regions are gated on and the MFI is reported for each antigen. Samples were run in duplicate per each secondary detection agent.

### Functional assays

Antibody-dependent monocyte and neutrophil phagocytosis was measured via a high-throughput flow cytometric assay^68,69^. Briefly, SIVmac gp140 was biotinylated with ﻿Sulfo-NHS-LC-LC Biotin (Thermo Scientific) following manufacturer’s instructions for 30 min and then washed with a ﻿Zeba Spin Desalting Column (Thermo Scientific, 87766) with 70 K MWCO. Yellow fluorescent streptavidin beads (Thermo Fisher) were coupled to biotinylated antigen and incubated with 1:100 diluted samples for 2 hours. Neutrophils were isolated from fresh donor blood with ACK lysis buffer (Thermo fisher) and added at a concentration of 5 × 10^4^ cells per well for 1 h at 37 °C. 2.5 × 10^4^ THP-1 cells (ATCC #TIB-202) were added per well and incubated for 16 hours at 37 °C. Neutrophils were stained with anti-CD66b (G10F5, Pacific Blue, BioLegend #305112) and neutrophil population was defined as CD66b+. A phagocytosis score was calculated for ADCP and ADNP as the percentage of bead-positive cells multiplied by the MFI of bead-positive cells and then divided by 10,000. For measurement of NK activation and degranulation, ELISA plates (Thermo Fisher NUNC MaxiSorp flat bottom) were coated with SIV mac gp140 (300 ng per well) at 37 °C for 2 hours, followed by blocking with 5% BSA overnight at 4 °C. 50ul of samples were added at a 1:50 dilution to each well and incubated at 37 °C for 2 hours. NK cells were isolated using RosetteSep (Stem Cell Technologies) from buffy coats from healthy donors. Per well, 5 × 10^4^ NK cells stained with anti-CD107a (H4A3, PE-Cy5, BD #555802), brefeldin A (5 mg/ml) (Sigma), and GolgiStop (BD) were added and incubated for 5 hours at 37 °C. Afterward, NK cells were fixed and permeabilized using Perm A and B solutions (ThermoFisher). Surface stains anti-CD16 (3G8, APC-Cy7, BD #557758), anti-CD56 (B159, PE-Cy7, BD #557747), and anti-CD3 (SP34.2, Pacific Blue BD, #557917) were added. Intracellular staining included anti-IFNγ (25723.11, FITC, BD #340449) and anti-MIP-1β (D21-1351, PE, BD #550078). Acquisition occurred by flow cytometry (iQue, Intellicyt). NK cells were defined as CD3- and CD56+, readout was % positive NK cells for each marker.

### SGA

Single genome amplification was performed to detect district proviral sequences from total DNA extracted from multiple tissue suspensions acquired following terminal necropsy^70^. Briefly, for plasma viral genome sequences, viral RNA was isolated and reverse transcribed to viral complementary DNA (cDNA) using the primer SIV GagPolR1 (5′-AATGGGGCACATAGCAAACC-3′). For DNA proviral sequences from tissue cell suspensions, total genomic DNA was isolated from enriched CD4 cells from each tissue analyzed using a negative CD4 selection magnetic bead kit, following the manufacturer’s instructions (STEMCELL). A limiting dilution series of 1:20, 1:40, 1:80, and 1:200 were made and for both cDNA and DNA samples, first-round PCR was carried out with Q5 High-Fidelity 2X Master Mix (New England Biolabs) together with primers SIV GagPolF1 (5′-AGTAAGGGCGGCAGGAACCAA-3′) and SIV GagPolR1. PCR conditions were programmed as follows: 1 cycle of 98 °C for 30 s, 35 cycles of 98 °C for 15 s, 55 °C for 15 s, and 72 °C for 3 min, followed by a final extension of 72 °C for 10 min. One microliter of first-round PCR product was added to Q5 Master Mix with primers SIV GagPolF2 (5′-CTATAAAGGCGCGGGTCGGTA-3′) and GagPolR2 (5′-TTATGAGGCTATGCCACCTCTC-3′). PCR conditions were programmed as above but increased to 45 cycles for the second step. Amplicons from cDNA and DNA dilutions resulting in less than 30% positive were considered to result from amplification of a single amplification and were processed for sequencing. For each sample, 15 to 30 sequences in total were analyzed using Geneious Prime 2020.2.2 software. Phylogenetic trees were generated using Figtree Vr.1.4.4 (http://tree.bio.ed.ac.uk/software/figtree/).

### Statistical analysis

Statistical analysis was performed using Graph Pad Prism 9 Vr. 9.0.0 for Mac (Prism, 2019) and license to the software was provided by the Barouch Lab. Linear regression analysis of setpoint viral load/peak viral load and percentage tissue viral DNA/viral RNA positivity was done using a simple linear regression model. Differences in viral DNA and viral RNA tissue positivity in Early ART study compared to Late ART study as well as cytokine response in LTs compared to GI compartments were performed using the two-tailed non-parametric Mann-Whitney test. Statistical significance was defined by a p-value < 0.05.

### Reporting summary

Further information on research design is available in the Nature Research Reporting Summary linked to this article.

## Supplementary information

Supplementary Information

Reporting Summary

## Data Availability

All data that support the findings in this manuscript are freely available from the authors upon request. Sequences have been deposited in GenBank with accession numbers MW473842-MW473939. Correspondence and requests for materials should be addressed to D.H.B. (dbarouch@bidmc.harvard.edu). Source data are provided with this paper.

## Source data

Source Data
