# Supplementary material for: Persistence of viral RNA in lymph nodes in ART-suppressed SIV/SHIV-infected Rhesus Macaques
Source: Nat Commun. 2021 Mar 5;12:1474. doi: 10.1038/s41467-021-21724-0 (PMC7935896; doi:10.1038/s41467-021-21724-0)
Supplement: Supplementary file 1 — Supplementary Information [file 41467_2021_21724_MOESM1_ESM.pdf]

Supplementary Information

Persistence of Viral RNA in Lymph Nodes in ART-Suppressed SIV/SHIV-Infected Rhesus Macaques

Anthony M. Cadena[1*], John D. Ventura[1*], Peter Abbink[1], Erica N. Borducchi[1], Hubert Tuyishime[1], Noe B. Mercado[1], Victoria Walker-Sperling[1], Mazuba Siamatu[1], Po-Ting Liu[1], Abishek Chandrashekar[1], Joseph P. Nkolola[1], Katherine McMahan[1], Nicole Kordana[1], Venous Hamza[1], Esther A. Bondzie[1], Emily Fray[2], Mithra Kumar[2], Stephanie Fischinger[3], Sally A. Shin[3], Mark G. Lewis[4], Robert F. Siliciano[2], Galit Alter[3], and Dan H. Barouch[1,3†]

[1] Center for Virology and Vaccine Research, Beth Israel Deaconess Medical Center, Harvard Medical School, Boston, MA 02215, USA
[2] Department of Medicine, Johns Hopkins University School of Medicine, Baltimore, MD 21205.
[3] Ragon Institute of MGH, MIT, and Harvard, Cambridge, MA 02139, USA; [3]Bioqual, Rockville, MD 20852, USA
[4] Bioqual, Rockville, MD 20852, USA.

[*] These authors contributed equally to this work

[†]Correspondence: Dan H. Barouch (dbarouch@bidmc.harvard.edu)

Supplementary Table 1: Mean log viral DNA and RNA copies per million cells in both secondary lymphoid tissues (LT) and gut for Late and Early ART cohorts.

	Viral DNA		Viral RNA	
	LT	Gut	LT	Gut
Late ART	1.09 (1.37)[1]	0.82 (0.45)	0.85 (0.00)	0.05 (0.00)
Early ART	0.46 (0.00)	0.57 (0.00)	0.15 (0.00)	0.03 (0.00)

[1]Mean (median) log viral copies per 10^6 cells

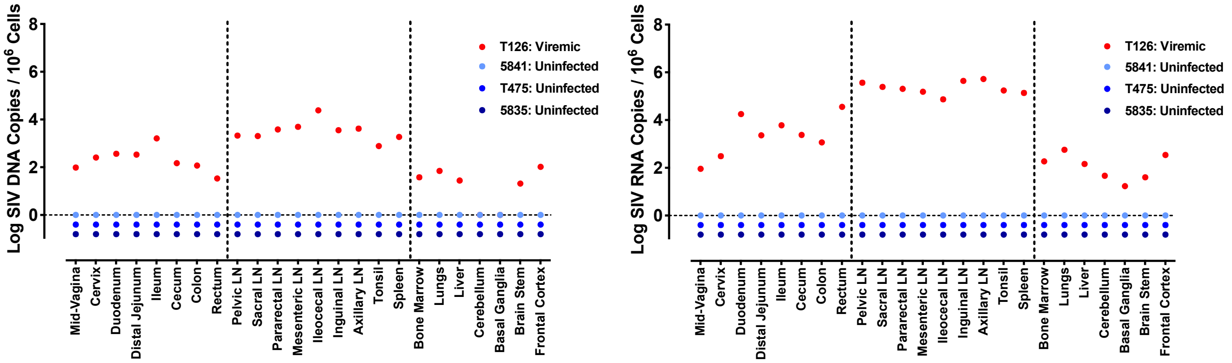

Supplementary Figure 1. Tissue viral DNA and viral RNA in practice necropsies. Tissue viral DNA (left) and viral RNA (right) are shown for 24 different tissues from 3 uninfected rhesus monkeys (in shades of blue) and 1 untreated, viremic rhesus monkey infected with SIVmac251 (in red). Each color represents a different monkey.

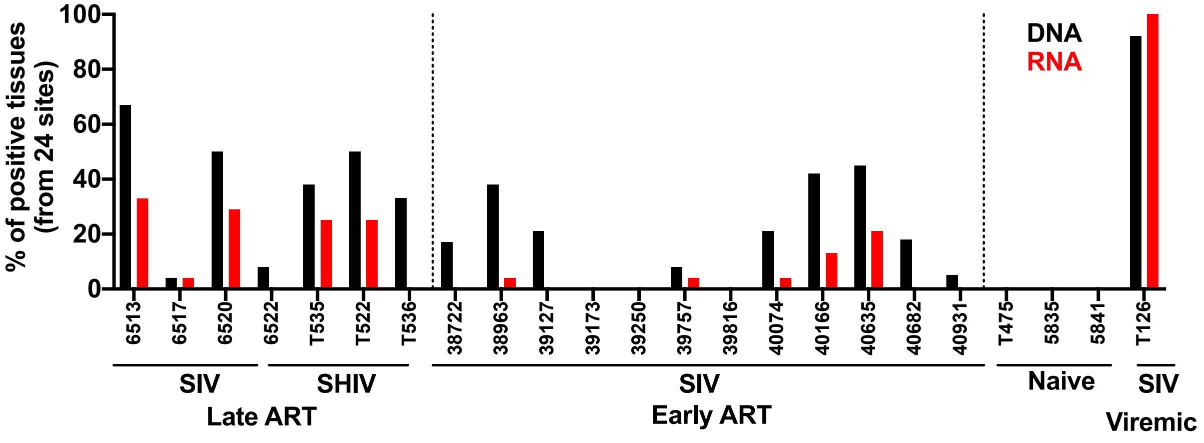

Supplementary Figure 2. Percent tissue positivity by study group. The percentage of tissues positive (out of 24) for viral DNA (black bars) or viral RNA (red bars) for the Late ART (left), Early ART (middle) and control monkeys (right). Each set of bars on the graph graphs represents one monkey.

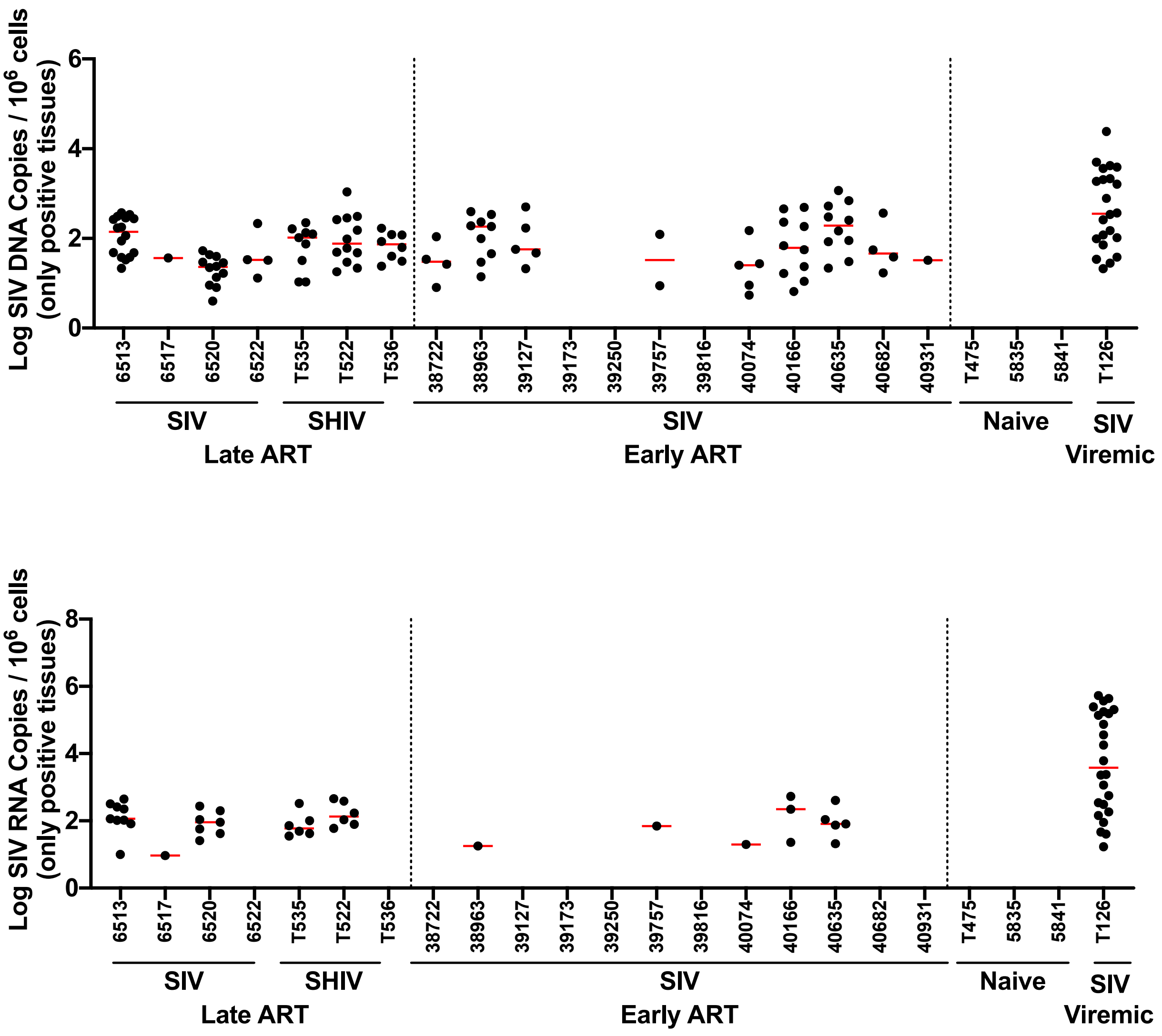

Supplementary Figure 3. Magnitude of viral DNA and viral RNA by study group. Levels of viral DNA (top) and viral RNA (bottom) for the Late ART (left), Early ART (middle) and control monkeys (right) (n = 24 tissues). Only positive tissues are displayed. Red bars represent median lines.

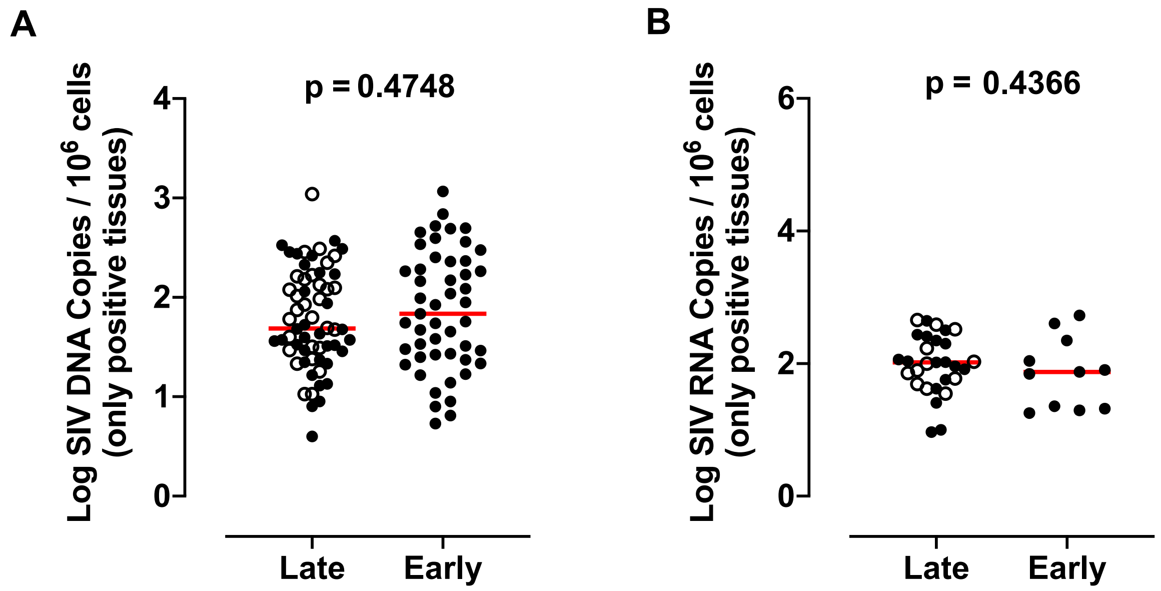

Supplementary Figure 4. Comparison of Late ART and Early ART viral DNA and viral RNA. (A) Viral DNA (Late ART, n = 62 and Early ART, n = 49) and (B) viral RNA (Late ART, n = 29 and Early ART, n = 11) of all positive tissues are shown. Each circle is a tissue and open circles denote tissues from the SHIV-SF162P3 animals in the Late ART study. p values reflect two-tailed Mann-Whitney tests. Red bars represent median lines.

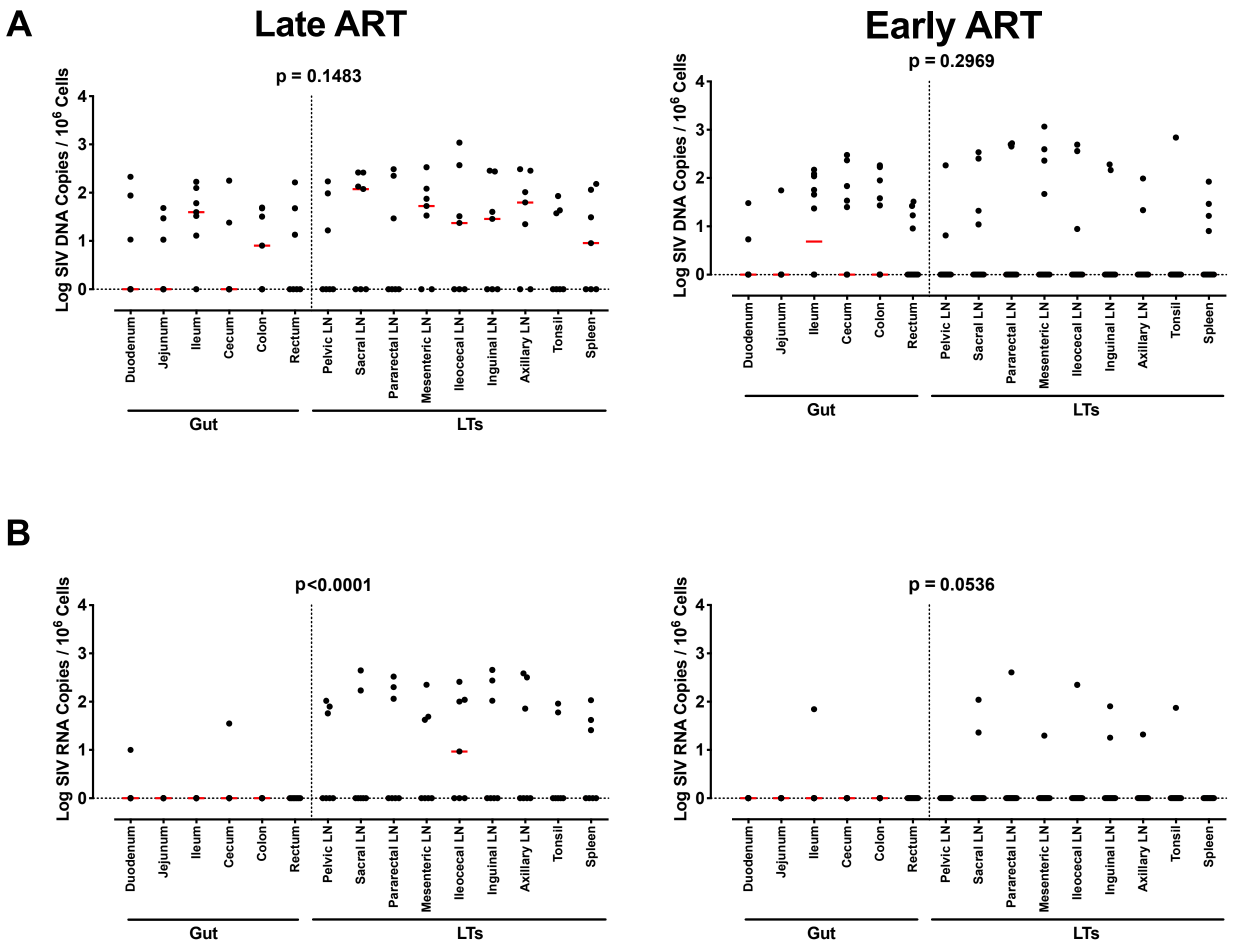

Supplementary Figure 5. Differential persistence of viral DNA and viral RNA in gut and lymphoid tissues. Comparisons of viral DNA (top) and viral RNA (bottom) for gut and lymphoid tissues from the Late ART (left, n = 7 animals) and Early ART (right, n = 12 animals) monkeys. Log SIV DNA copies/10^6 cells and log SIV RNA copies/10^6 cells are shown (Assay sensitivity was > 8-10 SIV DNA copies/10^6 cells and 10 SIV RNA copies/10^6 cells, respectively). Each dot represents a monkey, and red bars represent median lines. p values reflect two-tailed Mann-Whitney tests of binned tissue compartments.

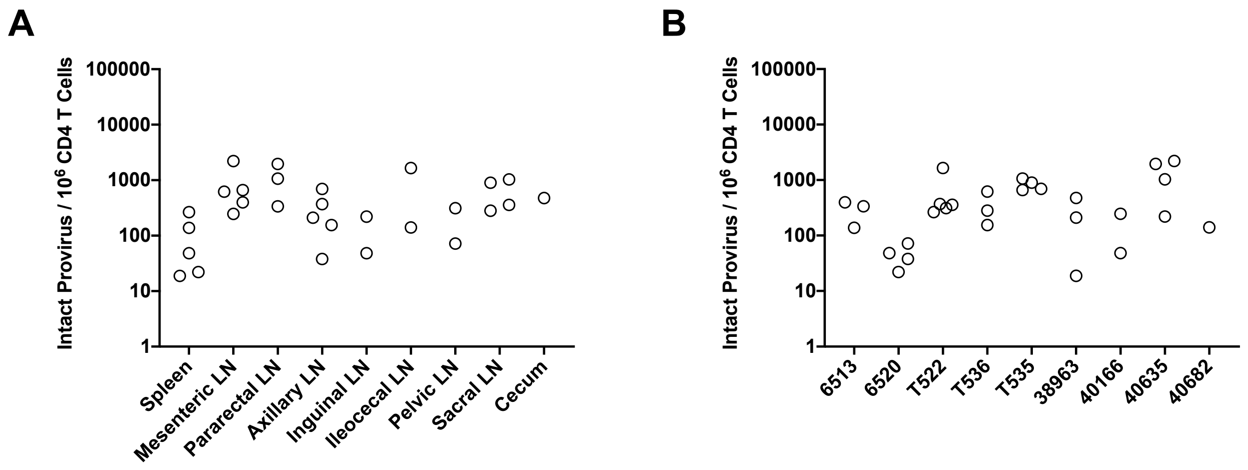

Supplementary Figure 6. Quantification of intact SIVmac251 proviral sequences in CD4+ T lymphocytes isolated from gut and secondary lymphoid organs at necropsy. Total quantification of intact proviral sequences per million CD4+ T cells via the intact proviral DNA assay (IPDA) distributed by tissue site (A) or by animal (B). Each dot represents either a separate tissue for (A) or a separate animal for (B).

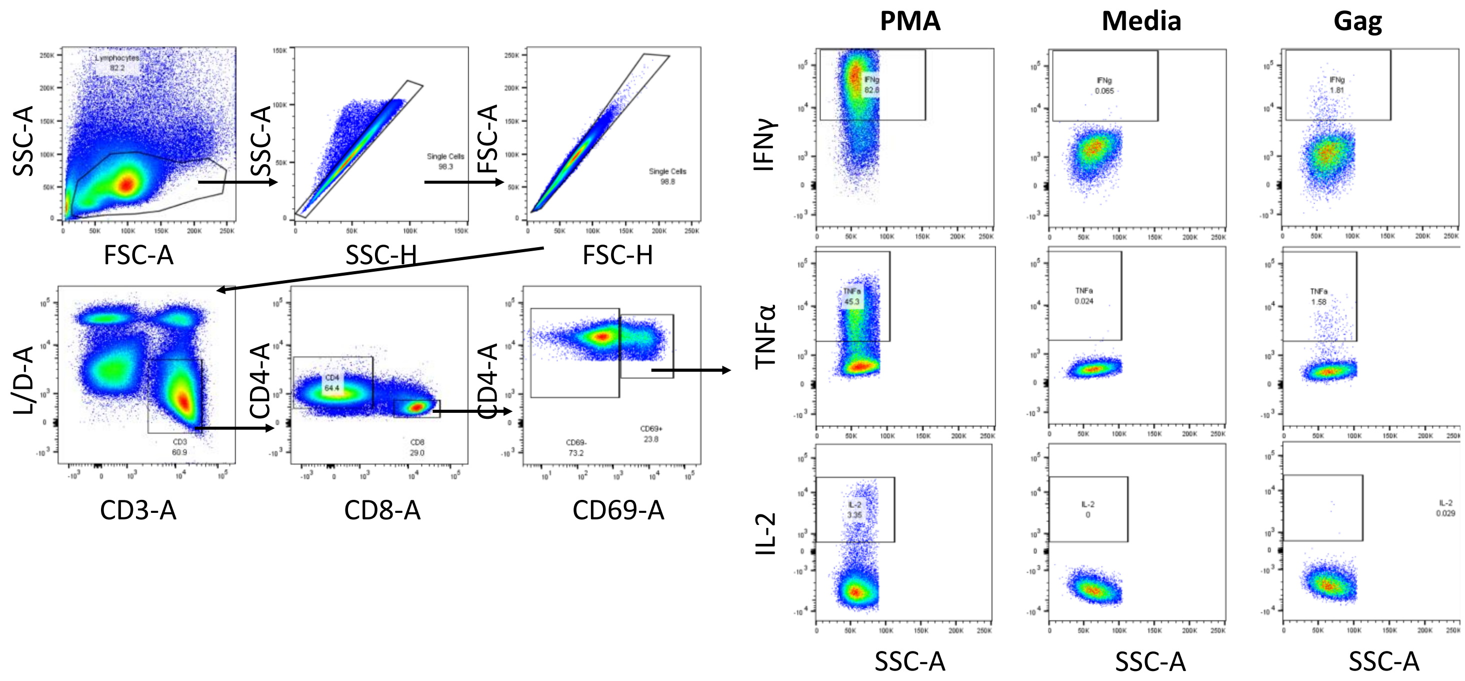

Supplementary Figure 7. Intracellular cytokine gating strategy. Representative gating strategy used to evaluate gag-specific cytokine responses of activated CD4 and CD8 T cells in Figure 5 in the main text and Supplementary Figures S8, S9, and S10. A negative control of R10 media and a positive control of phorbol myristate acetate (PMA) mixed with ionomycin were used as shown with representative CD4+ T cell responses for monkey T522. Tissues with less than approximately 2000 CD69+ T lymphocytes frequency were excluded from analysis. IFN-γ, Interferon gamma, IL-2, Interleukin 2, TNFα, Tumer necrosis factor alpha.

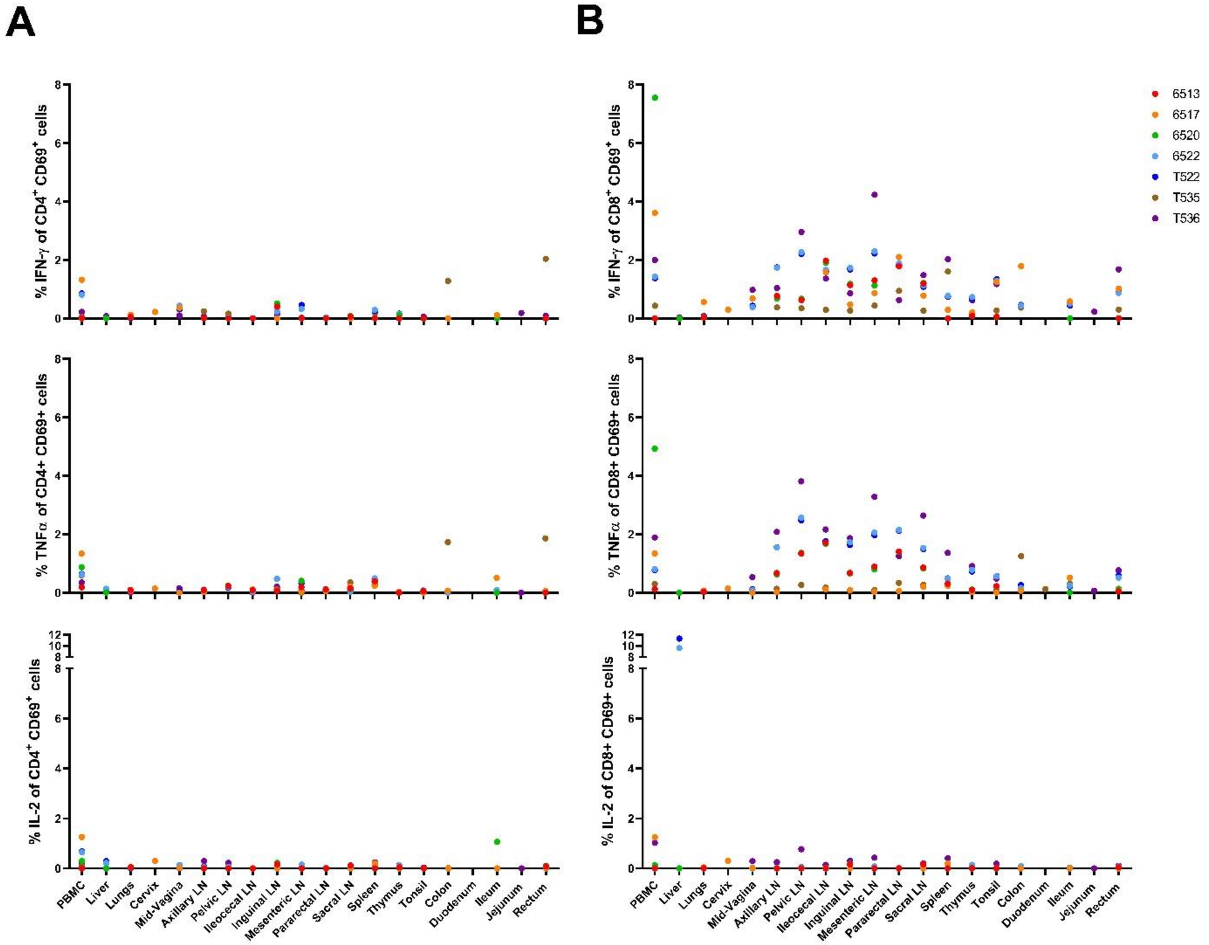

Supplementary Figure 8. Intracellular cytokine profiling of Gag-specific CD69⁺ CD4 and CD8 T lymphocytes in gut and lymphoid tissues from the Late ART group distributed by animal. Intracellular IFNγ, TNFα, and IL-2 expression from CD69+ CD4 (A) and CD8 (B) T lymphocytes form single-cell suspensions derived from gut and lymphoid tissues in the Late ART group collected directly at the moment of ART cessation. Different colored dots refer to individual animals and all tissues with approximately 2000 or more CD69+ T cells were analyzed. IFN-γ, Interferon gamma, IL-2, Interleukin 2, TNFα, Tumer necrosis factor alpha.

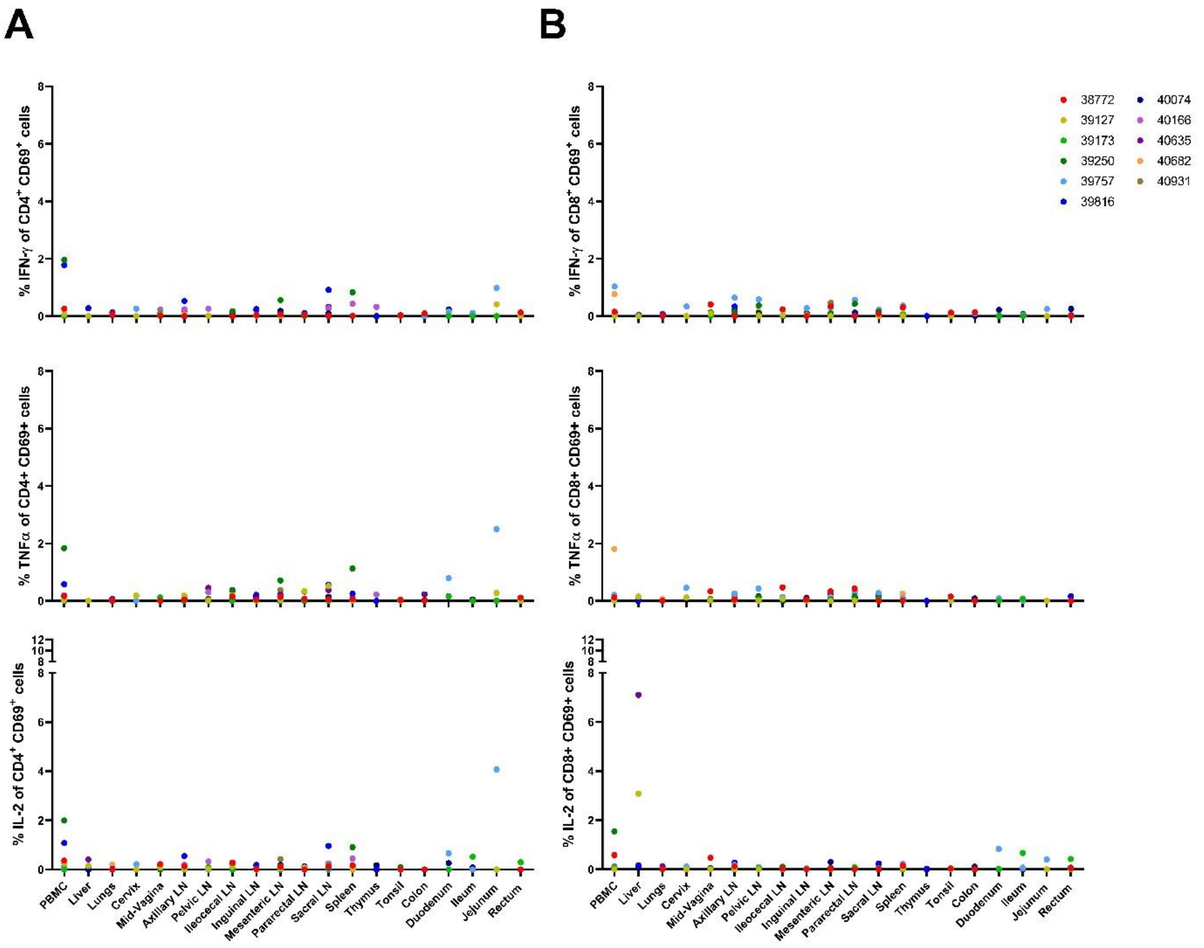

Supplementary Figure 9. Intracellular cytokine profiling of Gag-specific CD69$^+$ CD4 and CD8 T lymphocytes in gut and lymphoid tissues from the Early ART group distributed by animal. Intracellular IFNγ, TNFα, and IL-2 expression from CD69$^+$ CD4 (A) and CD8 (B) T lymphocytes form single-cell suspensions derived from gut and lymphoid tissues in the Early ART group collected directly at the moment of ART cessation. Different colored dots refer to individual animals and all tissues with approximately 2000 or more CD69+ T cells were analyzed. IFN-γ, Interferon gamma, IL-2, Interleukin 2, TNFα, Tumer necrosis factor alpha.

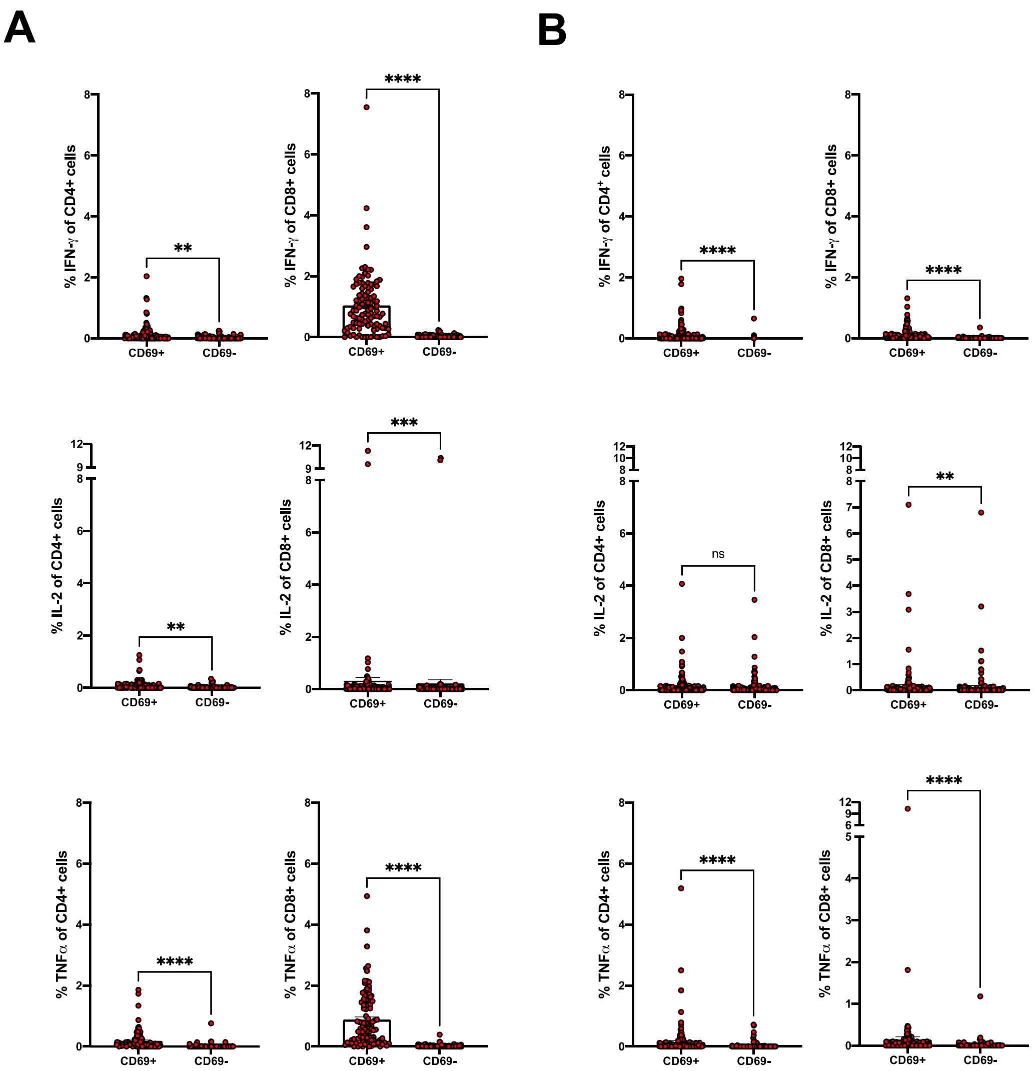

Supplementary Figure 10. Differential intracellular cytokine expression between CD69+ and CD69- CD4 and CD8 T lymphocytes. IFNγ, TNFα, and IL-2 expression between total CD69+ and CD69- CD4+ and CD8+ T cell populations from tissues analyzed in the Late ART (n = 103 tissues) (A) and Early ART (n = 153 tissues) (B) groups. Individual dots represent a tissue collected at necropsy and all tissues in aggregate were compared between CD69+ and CD69- populations. Statistical significance was calculated using two-tailed Mann-Whitney U tests. ****p = <0.0001, *** p = 0.0002-0.0001, ** p = 0.0021-0.0002, ns = not significant. Error displayed as the mean +/- SEM. IFN-γ, Interferon gamma, IL-2, Interleukin 2, TNFα, Tumer necrosis factor alpha.

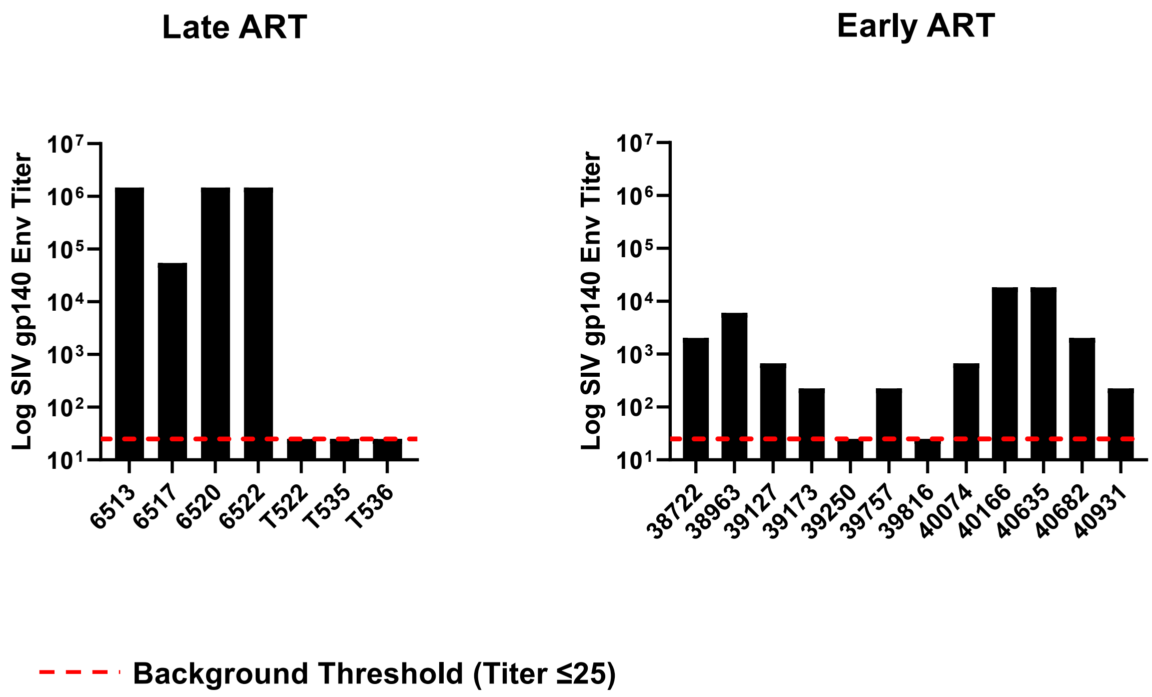

Supplementary Figure 11. Peripheral humoral responses in ART-suppressed animals. Serum log SIV gp140 Env titers from both the Late (left) and Early (right) studies are shown. Each bar is an animal. Dotted red line denotes the background threshold for gp140 (LOD = 25).

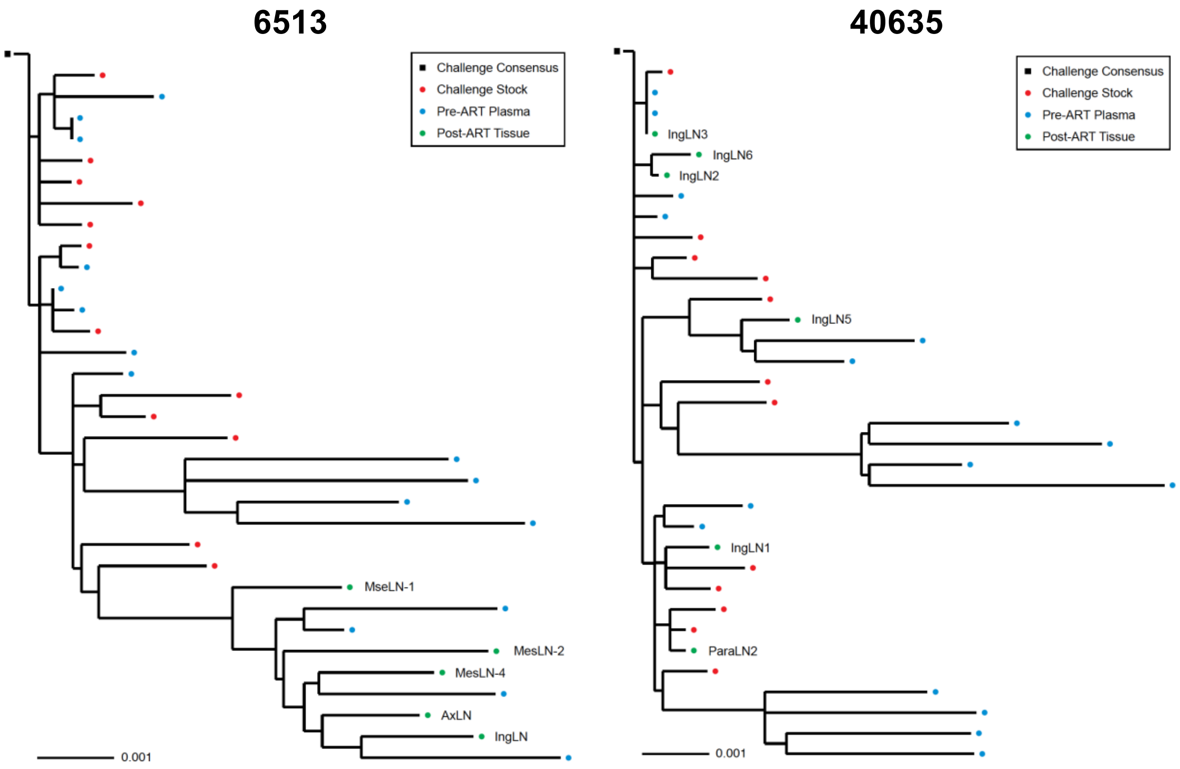

Supplementary Figure 12. Phylogenetic analysis of the *gag-pol* sequences of 2 animals infected with SIVmac251. *Gag-pol* sequences from Animals 6513 and 40635 via single genome amplification (SGA). Viral sequences were sampled at challenge (in red), were derived from pre-ART plasma (in blue), and from lymph nodes at necropsy during ART suppression (in green). The root of each tree is the consensus challenge stock (black square). MesLN, mesenteric LN, IngLN, inguinal LN, AxLN, axillary LN, ParaLN, pararectal LN.

Supplementary Table 2: Primer names and sequences

Name	Sequence	Assay
sGag21 (Forward)	GTCTGCGTCATCTGGTGCATTC	qRT-PCR Viral Load Assay / Viral Cellular DNA qPCR Assay
sGag22 (Reverse)	CACTAGGTGTCTCTGCACTATCTGTTTTG	qRT-PCR Viral Load Assay / Viral Cellular DNA qPCR Assay
sGag23 (Probe)	5' FAM -CTTCCTCAGTGTGTTTCACTTTCTCTTCTGCG-BHQ-3'	qRT-PCR Viral Load Assay / Viral Cellular DNA qPCR Assay
SIVmac251 IPDA *pol* forward	GCAGGGATAGAGCACACCTTTG	SIV IPDA
SIVmac251 IPDA *pol* reverse	CTATGGTTTCTACTGAATTTGCTTGTTC	SIV IPDA
SIVmac251 *pol* intact probe	5'-FAM-TTTCAGGTGGTGATTCA- MGBNFQ-3'	SIV IPDA
SIVmac251 *pol* hyper probe	5'-None-TAGGTGGTGATTTATT-MGBNFQ-3'	SIV IPDA
SIVmac251 IPDA *env* forward	CCTCAATAAAGCCTTGTGTAAAATTATC	SIV IPDA
SIVmac251 IPDA *env* reverse	GTTGTTGATGATTTTGTCAATCCC	SIV IPDA
SIVmac251 IPDA *env* intact probe	5'-VIC-TGCATTACTATGAGATGC-MGBNFQ-3'	SIV IPDA
SIVmac251 IPDA hyper probe	5'-None-TGCATTACTATAAAATGC-MGBNFQ-3'	SIV IPDA
rhesus macaque RPP30 forward 1	AGGATGCTCCGGGAGTATGTA	SIV IPDA
rhesus macaque RPP30 reverse 1	CCTGCTTGTCACCTATATAACAT	SIV IPDA
rhesus macaque RPP30 probe 1	5'-FAM- TCAAGCTGGGAGACGGAAGAGTCAGT- ZEN/IABkFQ-3'	SIV IPDA
rhesus macaque RPP30 forward 2	ACAGACTCACACAATTTAGG	SIV IPDA
rhesus macaque RPP30 reverse 2	ACATTCATGCCACTGCACTC	SIV IPDA
rhesus macaque RPP30 probe 2	5'-HEX- ACAGGGTCTCACTTTGTTGTCCA-ZEN/IABkFQ-3'	SIV IPDA
2LTR F	CGCCTGGTCAACTCGGTACTC	SIV IPDA
2LTR R	GGTATGATGCCTTCTTCCTTTTCTAAG	SIV IPDA
2LTR Probe	FAM- CCCTGGTCTGTTAGGACCCTTTCTGCTTTG-MGBNFQ	SIV IPDA
SIV GagPolF1	AGTAAGGGCGGCAGGAACCAA	Single Genome Amplification
SIV GagPolR1	AATGGGGCACATAGCAAACC	Single Genome Amplification
SIV GagPolF2	CTATAAAGGCGCGGGTCGGTA	Single Genome Amplification
SIV GagPolR2	TTATGAGGCTATGCCACCTCTC	Single Genome Amplification
