# Supplementary material for: Persistence of viral RNA in lymph nodes in ART-suppressed SIV/SHIV-infected Rhesus Macaques
Source: Nat Commun. 2021 Mar 5;12:1474. doi: 10.1038/s41467-021-21724-0 (PMC7935896; doi:10.1038/s41467-021-21724-0)
Supplement: Supplementary file 2 — Reporting Summary [file 41467_2021_21724_MOESM2_ESM.pdf]

nature research

Corresponding author(s): Dan H. Barouch

Last updated by author(s): Jan 17, 2021

Reporting Summary

Nature Research wishes to improve the reproducibility of the work that we publish. This form provides structure for consistency and transparency in reporting. For further information on Nature Research policies, see our Editorial Policies and the Editorial Policy Checklist.

Statistics

For all statistical analyses, confirm that the following items are present in the figure legend, table legend, main text, or Methods section.

n/a	Confirmed	
☐	☒	The exact sample size (*n*) for each experimental group/condition, given as a discrete number and unit of measurement
☐	☒	A statement on whether measurements were taken from distinct samples or whether the same sample was measured repeatedly
☐	☒	The statistical test(s) used AND whether they are one- or two-sided<br>*Only common tests should be described solely by name; describe more complex techniques in the Methods section.*
☐	☒	A description of all covariates tested
☐	☒	A description of any assumptions or corrections, such as tests of normality and adjustment for multiple comparisons
☐	☒	A full description of the statistical parameters including central tendency (e.g. means) or other basic estimates (e.g. regression coefficient) AND variation (e.g. standard deviation) or associated estimates of uncertainty (e.g. confidence intervals)
☐	☒	For null hypothesis testing, the test statistic (e.g. *F*, *t*, *r*) with confidence intervals, effect sizes, degrees of freedom and *P* value noted<br>*Give P values as exact values whenever suitable.*
☒	☐	For Bayesian analysis, information on the choice of priors and Markov chain Monte Carlo settings
☒	☐	For hierarchical and complex designs, identification of the appropriate level for tests and full reporting of outcomes
☐	☒	Estimates of effect sizes (e.g. Cohen's *d*, Pearson's *r*), indicating how they were calculated

Our web collection on statistics for biologists contains articles on many of the points above.

Software and code

Policy information about availability of computer code

Data collection	No software was used to collect data.
Data analysis	GraphPad Prism v9.0, Softmax Pro 6.1, QuantaSoft Analysis Pro 10.596, Geneious Prime 2020.2.2, and FigTree v1.4.4

For manuscripts utilizing custom algorithms or software that are central to the research but not yet described in published literature, software must be made available to editors and reviewers. We strongly encourage code deposition in a community repository (e.g. GitHub). See the Nature Research guidelines for submitting code & software for further information.

Data

Policy information about availability of data

All manuscripts must include a data availability statement. This statement should provide the following information, where applicable:

- Accession codes, unique identifiers, or web links for publicly available datasets
- A list of figures that have associated raw data
- A description of any restrictions on data availability

The data that support the findings of this study are available from the corresponding author upon reasonable request.
Sequence data that support the findings of this study have been deposited in GenBank with the primary accession codes: MW473842-MW473939

Field-specific reporting

Life sciences study design

All studies must disclose on these points even when the disclosure is negative.

Sample size	Sample size was N=44 animals (N=8 animals in the Late ART group; N=12 in the Early ART group; N=20 for long-term ART suppression group and N=4 practice necropsy animals). Based on our experience with SIV and SHIV in rhesus macaques (Barouch et al Cell 2016), this sample size can differentiate large differences between treatment groups. For all animal necropsies, we sampled 24 different tissues from each animal, which is a comprehensive array of tissues from multiple sites of potential viral reservoir.
Data exclusions	One monkey infected with SHIV-SF162P3 in the Late ART group died due to AIDS complications and was excluded from analysis. For the immunological analyses, a threshold of 2000 CD69+ T lymphocytes was used to eliminate frequency bias due to low cell number.
Replication	All attempts at replication of virologic and immunologic assays were successful. Multiple time points were assessed, where applicable, to ensure reproducibility. Virologic and immunologic measures were performed in duplicate. Technical replicates were minimally different.
Randomization	Animal expressing TRIM5 alleles were distributed between study groups. Animals were otherwise randomly allocated to groups.
Blinding	Immunologic and virologic assays were performed blinded.

Reporting for specific materials, systems and methods

We require information from authors about some types of materials, experimental systems and methods used in many studies. Here, indicate whether each material, system or method listed is relevant to your study. If you are not sure if a list item applies to your research, read the appropriate section before selecting a response.

Materials & experimental systems

n/a	Involved in the study	
☐	☒	Antibodies
☐	☒	Eukaryotic cell lines
☒	☐	Palaeontology and archaeology
☐	☒	Animals and other organisms
☐	☒	Human research participants
☒	☐	Clinical data
☒	☐	Dual use research of concern

Methods

n/a	Involved in the study	
☒	☐	ChIP-seq
☐	☒	Flow cytometry
☒	☐	MRI-based neuroimaging

Antibodies

Antibodies used	Antibodies were used for flow cytometry and ELISA assays.<br>Becton-Dickinson: CD3 (clone SP34.2; Alexa 700), CD4 (clone L200; BV7860), CD8 (clone SK1; APC H7), IFN-g (clone B27, PE-Cy7), IL-2 (clone MQ1-17H12, APC), TNFa (clone Mab11, FITC), CD28 (clone L293, PerCP-Cy5.5), CD95 (clone DX2, PE), CD16 (clone 3G8, APC-Cy7), CD56 (clone B159 PE-Cy7), IFN-g (clone 25723.11, FITC), MIP-1β (D21-1351, PE)<br>Beckman Coulter: CD69 (TP1.55.3, ECD)<br>Biolegend: CD66b (G10F5, Pacific Blue)
Validation	All antibodies were used per manufacturer's instructions and confirmed for reactivity for rhesus monkey samples.

Eukaryotic cell lines

Policy information about cell lines

Cell line source(s)	THP-1 cells
Authentication	Commerically purchased (ATCC)
Mycoplasma contamination	Negative for mycoplasma
Commonly misidentified lines (See ICLAC register)	N/A

Animals and other organisms

Policy information about studies involving animals; ARRIVE guidelines recommended for reporting animal research

Laboratory animals	M. mulatta, mixed male and female, age 3-8 years.

Wild animals	This study did not involved wild animals.
Field-collected samples	This study did not involve samples collected from the field.
Ethics oversight	All studies involving monkeys were conducted in accordance with the Harvard Medical Area Institutional Animal Care and Use Committee (IACUC).

Note that full information on the approval of the study protocol must also be provided in the manuscript.

Human research participants

Policy information about studies involving human research participants

Population characteristics	De-identified human PBMC were commercially purchased; no studies involved human research participants
Recruitment	None
Ethics oversight	*Identify the organization(s) that approved the study protocol.*

Note that full information on the approval of the study protocol must also be provided in the manuscript.

Flow Cytometry

Plots

Confirm that:

- [x] The axis labels state the marker and fluorochrome used (e.g. CD4-FITC).
- [x] The axis scales are clearly visible. Include numbers along axes only for bottom left plot of group (a 'group' is an analysis of identical markers).
- [x] All plots are contour plots with outliers or pseudocolor plots.
- [x] A numerical value for number of cells or percentage (with statistics) is provided.

Methodology

Sample preparation	Fresh cells were obtained from all sampled tissues through mechanical disruption of the tissues and through filtration of a 20uM mesh filter. Cells were stained for surface markers and intracellular cytokines according to manufacturer's protocols for flow antibodies. A negative control of R10 media and a positive control of PMA/Ionomycin was used to compare and establish gating for all monkeys.
Instrument	LSR II (BD)
Software	BD FACSDiva
Cell population abundance	Tissues with less than 2000 CD69+ T lymphocytes were excluded from analysis. See above for rationale.
Gating strategy	A representative gating scheme is provided in Fig. S7 defining the gating used for the tissues. Gating was determined by isolating distinct populations of antigen-specific lymphocytes from media-only control populations

- [x] Tick this box to confirm that a figure exemplifying the gating strategy is provided in the Supplementary Information.
